# Infertile women of Ethiopia: Psychological challenges and coping strategies

**DOI:** 10.1097/MD.0000000000037725

**Published:** 2024-04-12

**Authors:** Tinisaie Biadigie Adane, Kelemu Zelalem Berhanu, Abatihun Alehegn Sewagegn

**Affiliations:** a Mekdela Amba University, South Wollo, Ethiopia; b University of Johannesburg, Johannesburg, South Africa; c Debre Markos University, Debre Markos, Ethiopia and University of Johannesburg, Johannesburg, South Africa.

**Keywords:** infertile, psychological challenge, women

## Abstract

Worldwide, an estimated 48 million couples and 186 million individuals are infertile, according to estimates from the World Health Organization. Ethiopia has a higher rate of infertility than the World Health Organization estimated for the entire world. Though research on the issue of infertility is growing both globally and in Ethiopia, not much has been studied. Therefore, the main objective of this study was to explore the psychological and coping strategies of infertile women in Bichena town, Ethiopia. The study followed a qualitative research approach and a descriptive phenomenological design. Data were collected through in-depth interviews with 15 infertile women using a purpose-sampling technique. Thematic analysis was the method of data analysis. The findings of this study indicated that stress, anxiety, depression, low self-esteem, and sexual dissatisfaction were the main psychological challenges that infertile women experience. Infertile women also used a variety of coping strategies, such as religious strategies, traditional strategies, medical strategies, and other strategies (marital separation and acceptance). The study concluded that infertile women in the study area were challenged by psychological factors and used different coping strategies to manage their ongoing problems. This study also has theoretical implications for the current literature and practical implications for infertile women, non-governmental organizations, community and health professionals.

## 1. Introduction

The inability of a sexually active, noncontraceptive couple to become pregnant in a year is known as infertility, which is a condition of the reproductive system defined by the inability to become pregnant for 12 months or more with regular, unprotected sexual activity.^[1,2]^ Demographers define physiological infertility as an individual or a couple’s incapacity to reproduce. It is also known as infecundity, sterility, or physiological infertility. Men, women, both, or an unknown cause can be the cause of infertility.^[2]^ Female infertility can result from a variety of conditions, including endometriosis, polycystic ovarian syndrome, hormonal imbalances, early ovarian failure, genital infections, fallopian tube obstruction, congenital uterine anomalies, uterine synechiae, prolonged oral contraceptive use, sociocultural factors, and other medical complications.^[3]^ Moreover, infertility can result from issues with sexual life, lifestyle, and circumstances in general, as well as from physiological factors or issues with fertility.^[4]^

Globally, the prevalence of women’s infertility is increasing. Couples who will have trouble conceiving are estimated to be 15 million. One in 4 couples in developing countries is affected by infertility, and about 48.5 million couples experience infertility worldwide.^[5]^ In Africa, the prevalence rate of infertility can be as high as 20% to 30% in some areas and can vary from region to region even within the same country.^[6]^ The exact prevalence of infertility in developing countries has been reported to be unknown due to the lack of registration and the scarcity of empirical investigations in the area.^[6,7]^ In Ethiopia, it is difficult to get the prevalence of infertility. However, the rate of fertility has been highly decreased. For example, the fertility rate in 1950 was 7.331 per woman. It became a decrease and decrease and reached 4.014 in 2021(the growth rate is −2.310%), and in 2022 about 3.918 (the growth rate is −2.390%). These data do not directly indicate the prevalence of infertility. However, it shows the fact that women are highly exposed to the risk of being infertile.^[5]^

According to Akalewold et al,^[2]^ due to lifestyle changes and the presence of various environmental pressures, the prevalence of infertility has increased significantly and has become the third most serious disease after cancer and cardiovascular diseases. Furthermore, the World Health Organization estimates that between 48 million couples and 186 million people live with infertility worldwide.^[8]^ It can be classified as primary and secondary. Primary infertility is termed as if conception has never occurred, while secondary infertility means that the patient cannot conceive after having achieved a previous conception.^[1]^

According to the study by Halkola et al,^[4]^ infertility is often seen as a psychosocial crisis with strong emotions for the expecting child, and feelings often fluctuate between hope and despair. The study also assured that it can be associated with sadness, anxiety, stress, feelings of inferiority, failure, guilt, and fear of being left alone without a family. Studies analyzing women with infertility suggest that psychological problems such as depression and anxiety, excessive stress, emotional exhaustion, and hopelessness are common.^[9]^ A study conducted in Turkey by Karaca and Unsal^[10]^ has shown that infertile women suffer from various psychosocial problems due to infertility, such as negative self-concept, perceived social pressure, perceived social support, psychological symptoms, social withdrawal and isolation. Studies have revealed that women with infertility problems are often affected by depression, loss of self-esteem and self-confidence, and a general loss of control.^[2]^

Several empirical studies worldwide showed that women with infertility use a variety of strategies to cope with their stressors of infertility, which in turn are known to influence well-being.^[6,10–14]^ Infertile women have used traditional (cultural) treatment mechanisms or modern (medical) treatment mechanisms, or both, based on their preferences and exposures. Some coping strategies adopted by women with infertility may do more harm than good to them rather than strengthening them. Some may be beneficial for women and problematic for their partners.^[14]^ As a modern coping strategy, in vitro fertilization and embryonic transfer technology is an assisted reproductive technology and a sign of hope for fertility among infertile women.^[15]^ Other ways of coping strategies include distancing yourself from self-blame, self-controlling, and accepting responsibility.

There are many studies that discuss the challenges of being infertile women or men around the world.^[14,16–18]^ However, in Ethiopia, it is challenging to obtain well-documented data and recent studies related to the psychosocial and cultural challenges of infertile women.^[2]^ Akalewold^[11]^ conducted among married men and women in Addis Ababa, Ethiopia, regarding the psychosocial problems of infertility indicated that infertile women still practice traditional methods of healing infertility and are exposed to several problems, including incurring costs and practicing unwanted sex with traditional healers, such as wizards, to conceive a baby. In addition, due to the lack of awareness from the public, infertile couples, particularly women, are stigmatized and avoided social occasions. The study also stated that childless couples are subject to the critique of relatives and the community at large. Desalegn et al^[1]^ found that age at first pregnancy, age at menarche, multiple sexual partners, and number of days of menstruation flow were determinants of infertility of women. Based on the studies investigated above, the first gap identified in these studies is the scope of the content. Previously, in Ethiopia, several scholars^[1,11,12]^ have conducted investigations on infertile women focusing mainly on investigating the problems of infertility, magnitude, prevalence of infertility, determinant of infertility, sociocultural perceptions, and impacts of infertility on lived experiences of women who live in Addis Ababa, southern Ethiopia, and few parts of the Amhara Region, in Gondar and Dessie city. This study focuses mainly on women alone in order to explore their challenges due to being infertile from psychological dimensions. This study also incorporates coping strategies that help infertile women cope with existing challenges. Furthermore, the other gap identified in the above-mentioned studies is geographic scope, which means that almost all studies are limited in study areas. The studies are carried out in other parts of Ethiopia, not in the city of Bichena, and the women in the study area are selected from hospitals or clinics of governmental or private institutions who attended their medical care. This means that women who are infertile and cannot go to health institutions for medical support and instead prefer cultural and spiritual treatment are not studied. However, the current study bases the women on those who live in the city and may practice different treatment or coping strategies, which means they are not institutionalized. Therefore, the objective of the study is to understand the lived experience of infertile women related to psychological challenges and coping mechanisms in Bichena, Ethiopia. Moreover, to fill the gaps identified above and address the challenges mentioned above, the following research questions were raised.

1. What are the psychological challenges and experiences of being infertile women in the town of Bichena?
2. What coping mechanisms do infertile women use to deal with the difficulties associated with their infertility?

### 1.2. Theoretical framework

This study is supported by Scheper-Hughes and Lock’s three-body approach. The approach represents 3 levels of analysis^[19]^: the individual body (phenomenology), the social body (structuralism and symbolism), and the political body (poststructuralism). Scheper-Hughes and Lock do not see these levels of analysis as mutually exclusive but rather as interconnected. According to Scheper, Hughes and Lock (1987), as cited in Ofosu-Budu and Hanninen,^[19]^ the first level (the individual body) refers to “lived experience of the body-self.” The individual body experiences health, illness, happiness, or sorrow, although these experiences are influenced by social, political, and cultural factors. The second level of analysis is the social body (the body as a symbol).

Understanding viewpoints at the individual, community, structure, and dominant levels is made easier with the use of the three-body approach. It describes how bodies are perceived and given various meanings, what society views as appropriate or inappropriate, and how power and control are applied to the body. In order to connect the aforementioned method to this study, researchers first examine how infertile women feel about their condition and how they interpret its situation and treatments from a personal perspective. This is made possible by the notion of the individual body. Second, the social body plays an important role in capturing community members’ perceptions of the situation and treatments of infertility as well as the various psychological challenges these infertile women face. These perspectives help us understand how society views men and women in particular as being primarily to blame for reproductive failure. Lastly, the political body assists the researchers in looking at coping techniques.

The present study is also supported by the theory of social stigma. Stigma is a deeply discrediting attribute which reduces a whole person to a tainted and discounted other. According to Goffman, stigma can be put into 3 categories, namely physical differences, perceived character deficiencies, and tribal stigma of race, nation, or religion.^[19]^ The theory of social stigma is useful for this study to understand the challenges of infertile women living in a fertile community. In addition, this theory is also helpful for analyzing the cultural elements or practices of being infertile and how the cultural practices of the community look like to perceive, treat, or support infertile women and the challenges that infertile women have experienced. In a similar vein, there is a huge difference between individuals who are “fertile” and those who are not when infertility is viewed through the lens of social stigma theory. First, stigma of physical difference or deformation is an unfavorable opinion or attitude towards a characteristic of an individual or group of individuals connected to a perceived physical deficiency or infertility. It has to do with how a person or group is perceived negatively by society because of a perceived physical trait or attribute. There might be detrimental consequences on psychological wellbeing of women experiencing infertility. Second, stigma due to perceived character deficiencies refers to unfavorable views or opinions about other people based on distinguishing factors such as personality traits or actions that are seen as undesirable. As a result, the stigmatized group faces prejudice and a decline in status within the framework of social power, which in tun leads them to experience psychological distress. Third, the stigma associated with ideology is a negative attitude or idea about the characteristics of a person or group of people. When society or the general public share negative ideas, thoughts, or beliefs about women experiencing infertility, it affects stigmatized women negatively in terms of psychological aspects.

This study is also in line with the theory of coping. The theory was mainly used to measure the level of distress of people who experienced different social and health problems or people who faced stressful encounters such as mental health problems, breast cancer, infertility, and divorce. According to coping theory, in this study, coping is the continuous behavioral and cognitive efforts of infertile women to manage certain internal and/or external stresses that are thought to be stronger than the person’s resources. Individuals utilize their coping strategies as a tool to manage the obligations of life. The theory was primarily used to gauge the degree of discomfort experienced by those dealing with various health and social issues, or those going through difficult situations including infertility. According to Folkman et al (1986), coping serves 2 main purposes: first, it regulates unpleasant feelings (emotion-focused coping) and second, it modifies the problematic person-environment connection that is causing discomfort (problem-focused coping). The study followed a qualitative study approach. Therefore, it could not measure the distress level of infertile women. However, this theory enables researchers to understand the presence of distress in infertile women. The study has used coping theory to recognize and distinguish the coping strategies used by informants to solve external stigma and discrimination problems and internal emotional problems of their infertile women.

## 2. Materials and methods

### 2.1. Research design

The purpose of this study is to explore the psychological challenges and coping mechanisms of women experiencing infertility using their direct expression, words, feelings, opinions, and experiences of the phenomenon under study. Therefore, to achieve this aim, the use of qualitative research methodology was preferred during the study. Based on the purpose of the study, the study used a descriptive phenomenological research design. This is because according to Mayoh,^[20]^ descriptive phenomenology describes the common themes that experience, identity, phenomenon, and transcends the experience of a different individuals. The researchers chose a descriptive phenomenology because the study focuses on the description of the individual experience of the participants.

### 2.2. Participants

Bichena is a town in west central Ethiopia. Bichena is a town in west-central Ethiopia. Located in the Misraq Gojjam Zone of the Amhara Region on the hillside overlooking the Abay River, it has a latitude and longitude of 10°27′N 38°12′E and an elevation of 2541 meters above sea level. It is the administrative center of Enemay woreda (Wikipedia). Bichena is distant from the center of the Regional government (Bahir Dar) by 221.6 km, from the center of east gojjam zone (Debre Markos) by 90 km, and from capital of Ethiopia, Addis Ababa by 265 km. In 2022 the population of Bichena is about 65, 328 people. The town has made up 5 kebeles; (10, 421 people in kebele (Village) 01; 15, 123 people in kebele 02; 12,168 people in kebele 03; 16, 073 people in kebele 04; and 11,600 people in kebele 05). As seen from these 5 kebeles, the 2 kebeles (02 and 04) are more densely populated (Bichena city Administration Economic and Financial Development office, 2020). As the study is a qualitative approach, talking about the population and variables is not significant.^[21]^ Using a similar technique, 15 infertile women were sampled for this study. Infertile women in the 2 selected kebeles were identified with the help of urban health extension workers. They have better knowledge of the people who are in the vicinity. They have provided different health-related services to people at home. Thus, researchers have got infertile women when they are out from consultations with health extension workers and asked women’s permission to involve in the study.

The inclusion criteria set to select women in the study area to collect data through interviews and focus group discussions; age: the age of infertile women ranges from 18 to 49; who married for at least the last 12 months; who did not use contraceptives in the time 1 year before the date of the interview; women who had never been pregnant; who had not conceived in the past up to the date of our interview. The time it takes for an individual to become pregnant can vary. According to a study, if someone has stopped using contraceptives, research suggests that the time to pregnancy is between 2 and 3 menstrual cycles. However, around 90% of people will be pregnant within a year. The study found that about 30% of couples trying to conceive will become pregnant within the first month, and around 90% will do so within the first year. Couples in the present study take longer than this to get pregnant.^[6]^

### 2.3. Instruments

This study used in-depth interviews to collect data. This is because it enables researchers to get in-depth information about the experience of women in the study area. Therefore, based on the review of various articles in the literature, researchers identify indicators or develop semistructured questions or guideline items that are used to identify the psychological challenges and coping mechanisms of women with infertility. That means that the questions are prepared from reviews in the literature based on the main purpose of the study and the basic questions of the study. The main questions were: What are the psychological challenges and experiences of being infertile women in Bichena town? What coping mechanisms do infertile women employ to deal with the difficulties associated with their infertility? The duration of the interview was 25 to 30 minutes. Since researchers are open to new ideas and discoveries and have some background knowledge of the subject, the setting, and the target population, they conducted interviews. In addition to being polite, moral, and reflective, researchers also have certain abilities in listening, asking probing questions, and developing relationships. They were validated by experts in the area.

### 2.4. Data collection procedures

After getting an ethical approval, semi-structured data collection guides are translated from English to Amharic versions; the researchers visited the village of each infertile women of the study. The data were then collected through in-depth interviews with infertile women assigned for this. Next, the raw data collected through in-depth interview are then coded or broken down into manageable words, and the codes are translated into English. As a result, preliminary codes, followed by final codes, are developed. To perform thematic analysis, the coded data are examined using thematic coding.

### 2.5. Data analysis

The data collection, data analysis, and report writing processes are not distinct steps in research, are rather interrelated and go on simultaneously.^[22]^ The purpose of this study was to explore the psychological challenges and coping mechanisms of infertile women. Thus, in this study, qualitative thematic analysis was performed as a data analysis method. Rather, they were organized and complied based on similarities and differences, so common themes and subthemes were created that helped to analyze thematically. In accordance with the primary research questions, the data was examined. The researchers conducted interviews in Amharic before translating and transcribing what was discussed. The researchers double-checked the audio recordings to make sure that the translation and transcription were accurate because they were also familiar with the Amharic language. To become familiar with the entirety of the interview response and to detect themes; researchers listened to audio cassettes that corresponded with transcripts. Under psychological challenges (the first major theme), there are subthemes of stress, anxiety, depression, low self-esteem, and sexual dissatisfaction. The treatment strategy is a second major theme with subthemes of religious treatment strategies, traditional treatment strategies, medical treatment strategies, and other treatment strategies. These main themes and subthemes were properly analyzed.

### 2.6. Transferability and trustworthiness of the findings

Qualitative validity means that researchers check the accuracy of qualitative research and determine whether the findings are accurate from the point of view of researchers and women in the study area.^[22]^ In this study, the transferability and trustworthiness of the findings were achieved through the validation of emerging codes and categories in subsequent interviews. In addition to this, to determine the trustworthiness of the qualitative findings, the recorded data are checked twice to avoid obvious mistakes during the transcription process. Furthermore, the selection of women in the study area is not exhaustive, but the participant’s selection is carried out by setting inclusion and exclusion criteria before data are collected to avoid bias and subjectivity. As ethical considerations, after receiving ethical approval from institute of education and behavioral science research committee, researchers obtained written informed consent from each study participant after explaining the objectives and benefits of the study. After receiving written informed consent from women in the study area, they were ensured confidentiality and informed that participation is voluntary and that they can withdraw from the study at any time.

To determine if experts agreed on themes, interrater agreement using Cohen’s Kappa was examined. Twenty percent of the interview data were selected at random by the authors, and then impartial specialists recorded the data. The result showed.90. It is acceptable that showed experts agreed on the original coding data and the re-coding data.^[22]^ An audit trail was also kept in order to verify that the study’s conclusions are based on participant narratives rather than the author’s possible personal narratives.

## 3. Results

### 3.1. Demographic characteristics of the respondents

This study has generally employed 15 infertile women who participated in in-depth interviews. Eleven of them are followers of the Orthodox Christian religion and 4 of the remaining infertile women are Muslims. Most of them are literate (2 of them are degrees, 5 of them are diplomas, 5 of them are under grade 8 and 2 are illiterate). Except for 2 women, all have marital status. All of them have siblings and their siblings had born at least 1 child. Related to occupational status, 8 of them are civil servants, 4 of the infertile women are merchants, and the remaining 3 women are house wives. Of 4 Muslims, 3 of them are under polygamous marriage. Nine of the husbands of infertile women have children from previous wives or hidden women.

### 3.2. Psychological challenges and coping strategies of infertile women

**Figure 1.**
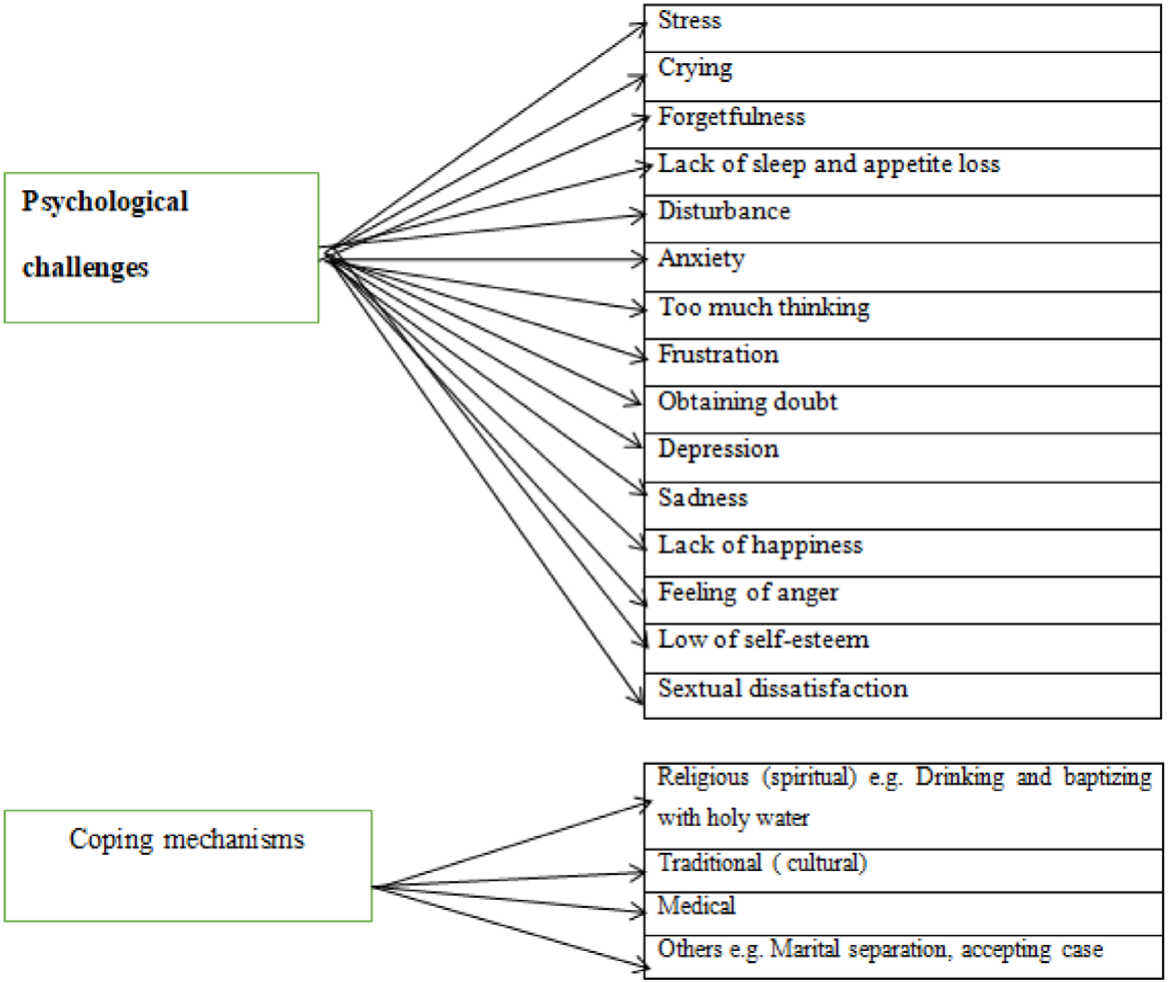
Details of themes and subthemes.

The semi-structured interview questions guided the analysis. Infertile women’s responses to their psychological challenges and coping mechanisms are presented in Figure 1

As shown in Figure 1, the women interviewed have used different coping mechanisms that address their psychological challenges and social capital. The details are presented as follows:

### 3.3. Psychological challenges

The first identified theme with respect to infertile women was psychological challenges. Infertile women who grew up in the town of Bichena were exposed to different forms of psychological challenges. The most commonly identified challenges are stress, depression, anxiety, low self-esteem, and sexual dissatisfaction.

### 3.4. Stress

‘Overall, *I am the house of stress’ (respondent*)

Infertile women have experienced psychological stress that can be expressed in different forms. Infertile women in Bichena city have been challenged by stress in the form of crying, forgetfulness, disturbances, and lack of sleep.

#### 3.4.1. Crying.

*‘My eyes are going to melt due to crying’ (respondent*)

The majority of infertile women have experienced stress and expressed their feelings when crying for different reasons. They have been abused by their husbands, family members, mother/father in law, colleagues at work, neighbors, and other members of the community in a different way.

For example, an infertile woman has fed her stress through crying due to community abuse. She shares her feelings as:

*Even I cannot express my deepest sorry for the people who have died during funeral ceremonies in the church. Those around me whispered, talked, and looked at me longer while I sobbed. They told me that since I couldn’t conceive, I should cry for myself. When I die, no one will be able to cry for me. I cried a lot after listening to them for a longer period of time. You know what I mean* (In-depth interview participant (IDP) 5)

#### 3.4.2. Forgetfulness.

Some infertile women in Bichena have been stressed due to their infertility problem and are exposed to the challenge of forgetting. *IDP5 and IDP8* have shared their stress in the form of forgetfulness as follows:

*When I think about my case of infertility when I am alone, I have been stressed for a longer time. Following this, when I need to do certain activities, I miss something. For example, at home a few days ago when I was cooking food, I missed some of the elements or ingredients or spices even lacking the order of preparation. I have shamed myself (IDP11*).

In addition to the above, reflection of feelings by *IDP* 9 and *IDP* 7 has remembered the case of forgetting that she had faced a few months ago after holding over stress due to her infertility problem. The situation made them stress more which finally made them forget things easily. Therefore, the above respondents said that due to their thinking about their infertility problem, they are exposed to forgetting different routine activities and those they create a certain burden on their daily life.

### 3.5. Lack of sleep and appetite loss

*‘My eyes long for a healthy sleep’ (respondent).*

Due to infertility, some infertile women in Bichena town have experienced a feeling of stress due to lack of sleep and consequently have also failed to eat properly. The following infertile women have shared their feelings of stress as follows.

I am not working at the moment. Now I am a housewife. When I am alone, I often think a lot about my case (infertility). After going to bed at night (with my husband), my husband goes to sleep easily after lying in the bed. However, sleeping is not that easy for me. I am lying on my back and steering on the roof. I capsize to the right and to the left again and again. Sometimes, my husband wakes up and says ‘do not disturb me.’ I am tired. I need rest. I don’t sit and sleep all day at home. So, the other time when I am asleep, I worry a lot not to disturb my husband (IDP12).

#### 3.5.1. Disturbance (lack of concentration).

*‘My mind easily tendered to a few negative words’ (respondents).*

In addition to crying, forgetfulness, and lack of sleep, other infertile women, on the other hand, described the feeling of stress as disturbed.

*My mother in law is an invector woman. Every activity of her and her sayings is full of jokes. She has been complaining, why am I not pregnant? I explained to him, but he couldn’t want to understand my explanation. Especially during the coffee ceremonial time, her talking is very disturbing to me. She always praised her other daughter-in-law, who has had children. She said that they are real wives. Such things are very disturbing to me and I experience stress. (IDP14).*

All the respondents above forwarded that because of infertility they have been exposed to different forms of stress. They have expressed their stress through crying, forgetting, lack of sleep, and disturbing. However, infertile women in Bichena town are not challenged by psychological stress. They have also been challenged by other forms of psychological challenge.

### 3.6. Anxiety

Infertile women in the study area were experiencing anxiety through frustration, doubtfulness, too much thinking, and other forms of psychological problems. These problems are identified as anxiety. Therefore, infertile women in the study area are challenged by anxiety and have expressed themselves in different ways which are mentioned above. The study aims to analyze different ways of expressing anxiety by infertile women.

#### 3.6.1. Too much thinking.

*‘I cannot stop my mind: it thinks too much’ (respondent*)

Some of the infertile women in Bichena have experienced too much thinking and reflect on different occasions. IDP 10 has shared her story as follows: “Even when I tell myself Okay, I’m not going to worry,” it is always on my mind. I cannot get it out of my mind. I always try, but I cannot stop my mind”. IDP15 also shared her feelings as follows:

*When I am alone, I start to think more about my case of infertility. Different ideas come to mind. In fact, there is no day I will not think of the situation that I am facing. I will be thinking, that is, how life will continue with me? Is that how I am going to end?*

Infertile women explored that they easily go into deep thoughts about their problems and their future life. They feel anxious about the conditions they have faced.

#### 3.6.2. Frustration.

Frustration is an aspect of anxiety that affects some of the women with infertility. Some infertile women in the study area have expressed their anxiety through frustration. They fear being infertile for different reasons. The frustration varies from being lonely, divorced, to their husband marrying a second wife. For example, IDP9 shared their frustration feeling as follows.

*At the moment, he (his husband) refused to tell me about the ongoing situation (my infertility). However, I think in my mind he will be hoping to marry another wife and I know one day that he would not be able to resist it. Whenever a woman calls him, I will be so anxious to hear their conversation and to identify who she is. Hmmm! I am so frustrated because I know that one day she will come.*

#### 3.6.3. Obtaining doubt.

Doubt is another means of expressing anxiety. The following women in the study area have shared their story about their doubts. *IDP*3 has expressed her doubts about the deterioration of the loving bondage and the status of marriage.

*From the beginning, my husband and I have a strong emotional bond. We strongly love each other. I believed that I cannot stand and lead my life without him and that he can also feel like me. However, recently, he (her husband) came home from work at night. I have observed a new behavior that was not before. He drinks a lot of alcohol. I have heard that he talks to another woman in a loving way. He can forget me in bed. So, I think our marriage life or continuity is in doubt. (IDP3*)

Therefore, infertile women have been challenged by anxiety and have expressed themselves in different ways. The common way to reflect your anxiety is too much thinking, frustration, and doubt. For these 3 ways of expressing anxiety, they stand for different reasons that were explained above. Infertile women have doubts about themselves and family members about the need to have children. Infertile women who have tried different solutions for born children and failed to achieve what they need experience doubt in themselves. On the other hand, infertile women who lack adequate attention from family members are in doubt of supporting them to try different forms of solutions for born babies.

### 3.7. Depression

Depression is another psychological challenge facing infertile women in the town of Bichena. According to the collected data, these infertile women are not challenged by psychological factors. They are also exposed to different social and cultural challenges. They have also undergone different treatment strategies. They have expressed that the inability to find a solution and become pregnant and the combination of different challenges caused them to experience depression. Infertile women in the study area are experiencing depression and have described their situation in different ways.

#### 3.7.1. Sadness.

*‘I feel like a hopeless and helpless woman’ (respondent*)

*IDP5* has described her reason for feeling sad as follows;

*Sometimes my school colleagues ask me: why are you sad? Is there any new problem? What happened? I said no. However, to be honest, I always look sad and my heart was suffering due to the case you know (her infertility). Because of that, I have preferred to stay alone doing other things (IDP5*).

Some other infertile women also report feeling sad for the reason that when they hear children calling their parents. This reminds them of their failure to have children. *IDPS 1, 4, and 7 have reflected their feelings as follows;*

*I hear children calling their parents... mama, dad... when they go home and find something. Surprisingly, I have also heard their love conflict (children with their parents). I think in depth about my story and find it empty. So I feel very sad (IDP1, 4, 7).*

#### 3.7.2. Lack of happiness.

Some infertile women in the study area have also expressed their feelings of depression as they lack happiness in their life due to infertility. Infertile woman described that happiness is not easy for them.

*My mother in law and father in law said to me, why are you concerned about maintaining your hygiene? Your dressing is also good as we have seen. But all such activities are not a guarantee of your marriage. The only option is to become pregnant and have children born. So, do you expect happiness in such situations? They know how I tried to solve my problem. But they did not consider that and only worry about their son. Totally, happiness went away from me (IDP15*)

#### 3.7.3. Feeling of anger.

Some infertile women have also described their feelings of depression in the form of anger as follows. IDP14 stated “Sometimes, I don’t know the real reason for that particular moment. Ehhh! My God! I will be angry without even a cause. Every activity I have seen at home also caused me to be angry.” IDP12, also added her feelings as:

*When I go to ask women who were born, other fertile women are insulting me, talking behind me. Even if I did not clearly hear and understand what they have said, I guess it will be on my case (her infertility). In that situation, I get upset! Upset! Upset! Ohhhhhh! I immediately leave her house (the woman who had died) and go back to my home with anger. I don’t want to say more.*

### 3.8. Low self-esteem

One of the infertile women has expressed her feeling of inferiority due to her inability to bear children. IDP5 expressed her feelings “I always think that because I cannot get pregnant, I cannot have children, I am less than others. This idea really bothers me.” IDP1 also stated her self-esteem as “I am not comfortable with different parties involved at all. I don’t have a good feeling. My self-esteem really decreased. I don’t want to be among the others. I feel like I am boring compared to them.”

In addition, other infertile women have described their self-esteem status as jealousy and sensitivity;

*I became very sensitive. My brother’s wife became pregnant. She is younger than me and married after me. I became jealous of her and didn’t want to see her during her pregnancy time and at the time of delivery. I have created my own reason not to see her. I have compared with her negatively. I know it is inappropriate but I did not stop it*. (*IDP4*)

Respondents have shared that due to exposure to stress, anxiety, or depression, their life is not going better like that of fertile women that lower their self-esteem.

### 3.9. Sexual dissatisfaction

Regarding sexual dissatisfaction, the researcher has obtained 2 contradictory findings from the respondents. On the one hand, respondents described that they have no problems or lack in their sexual interest and that it continues as normal. Instead, their interest and frequency of having a sexual relationship increase after learning of infertility. On the other hand, after knowing about infertility, interest from the husband becomes decline. The husbands also humiliate and insult their wives. Consequently, the interest of women towards sexual intercourse is lowered. The study has confirmed these points with the response of the respondents.

*My husband requests sexual intercourse more frequently than before. My interest does not decline. Rather, I need to have more than usual. To get pregnant, I think frequent sexual intercourse is necessary. I also do for my husband. (IDP11*)

Infertile woman who was divorced has described as having an interest in sexual intercourse and going out with different men but is not sure of her satisfaction (IDP6).

To sum up, infertile women in Bichena city are suffering from different components of psychological challenges that are stress, anxiety, depression, low self-esteem, and sexual dissatisfaction. These identified psychological components exist either 2 or 3 of them in infertile women and one of the psychological components affects the infertile women to experience another one based on their response.

### 3.10. Coping strategies of infertile women

Infertile women in the study area have attempted different coping strategies for their infertility challenge. They used religious (spiritual) coping strategies, traditional (cultural) coping strategies, marital separation, accepting, and medical coping strategies. The most widely used coping strategy is traditional and religious strategies over medical ways. Some infertile women have tried to use medical coping strategies, but have returned to traditional and religious methods.

a. Religious (spiritual) coping strategies

‘If *you are religious, your womb is full of babies’ (respondent).*

‘God *helps those who help themselves’ (respondent*)

According to the respondents, a religious coping strategy is preferable to solve their infertility challenges. This is because all options, including medical treatment, can become effective when God allows this to happen. Therefore, they prioritize this strategy.

#### 3.10.1. Drinking and baptizing with holy water.

Some infertile women have described that they have applied religious strategies to solve their infertility problems. They have gone to different places in the country that have “Tsebel” Holy water with practical miracles. According to their response, they have received the names of holy water and places with great miracles. Therefore, they go to holy water, which cures the infertility problem.

*In addition to medical treatment, I have tried holy water. My neighbors told me about a holy water at Ba’eta found in North Gondar. That holy water is known to solve the infertility problems of a 50- year woman who wants to give birth. When I drank the holy water, something like a small fish came out of my stomach. However, I did not get children* (IDP5).

Another infertile woman has also validated that she went to the “*holy water of Saint Lalibela”* which was found in the Amhara region, the northern Wollo area and never solved her problem, even if she tried many times to visit that holy water. IDP2 stated as follows “Some infertile women said that they could not solve their infertility problem with medical treatment, rather by baptizing and drinking holy water in ‘Saint Lalibela holy water’. However, that holy water did not work for me.”

#### 3.10.2. Advice and prayer of the confession father and religious leaders.

Some other infertile women have explored that they have told their confession to the father about their infertility problems and how much they find it difficult to have children and pray for them. They also mentioned that their confession fathers have given advice to them by picking some biblical characters who were infertile and got children after longer years of tolerance and strong belief in their God.

*I am not clean at all; why has God punished me. Every day, in the morning, I go to church to pray and pray for God. But all of my efforts are not reached to God. So, I have given my case to my confession father, which gives some relief to me, and one day God may listen to the confession father and give children for me. (IDP3*)

b. Traditional (cultural) coping strategies

Infertile women also replied that they have used different traditional or cultural strategies to alleviate their infertility problems. Traditional methods commonly tested are visiting traditional physicians (herbal healers) and going to wizards. An infertile woman has gone to a wizard who is found in south Wollo, Dessie town, and stayed with him for 3 months until her problem was cured. She said that after 2 weeks of treatment, her stomach became wider and bigger and the wizard told her that she got a pregnancy and after 9 months had been born a child. However, she could not have a child in addition to exposing for stomach pain.

Some infertile women also go to herbalists (traditional doctors) who treat different diseases by applying plants. Most of the time, these herbalists are called “Merigeta” and they advertise their work on a billboard as “traditional doctor or physician of Merigeta.”

c. Medical coping strategies

Some infertile women have described that they tied medical treatments in different places to solve their infertility problem. However, some of them have stopped the treatment process as they could not find new members, and others leave due to financial deficits.

Emebet, Aychilushim, yamrot, and Senait have all explored that they have followed the medical treatment process for longer years in Addis Ababa, Bahir Dar, and Debre Markos. But they could still not become pregnant and they had children.

*I have spent my summer at Addia Ababa Tkur Anbessa Hospital. I have taken the drug and my womb is washed and cleaned. (IDP5*)

Key informants who are health professionals said that infertility is a global problem for both men and women and can be treated. They described that some women have to go to their hospital and consult about their problems and then try to help them based on their problems.

### 3.11. Other ways of coping strategies

In addition to religious, traditional, and medical coping strategies, infertile women also used other means of coping to solve their problems. These methods cannot directly solve their problems and allow them to have children. However, some strategies reduce pressure, stress, and stigma from other people.

### 3.12. Marital separation

Two infertile women have said that they have been exposed to different forms of family pressure and abuse from the husband and his relatives. They tolerate for longer years to continue their marital status alive. However, when it became very ugly and they cannot cope, they have decided to break their marital status voluntarily.

*I tried a lot to solve my infertility problem. My husband, my mother-in-law, and all my husband’s relatives clearly know about my efforts. However, they blamed me and stigmatized me blindly. No one understands me. Therefore, it was better to divorce and continue as a single. Currently, I have avoided mocking and insulting my former husband and his mother. I continued to search for other options freely*. (*IDP* 5 and *IDP 7*)

### 3.13. Accepting case

Some infertile women have used another strategy to cope with their infertility problem, accepting the truth. They mentioned that they have tried different strategies, traditionally, medically or religiously, and nothing will happen. Therefore, the only thing is to accept the problem as natural and focus on your life without children.

*Of course, it is bitter to live without children. But it is beyond my power. I have tried to exercise living alone. My husband supports me. But I have told myself that whatever happens, I can resist the coming challenge and continue my life as normal. I said that it is not the only problem that happens to me in the world. So, I can live. Children are not allowed in my house. I convince myself. (IDP15*)

Infertile woman reported that they believe everything is predetermined by nature and that human beings have a very limited chance of changing what God determines as they expressed their ideas.

## 4. Discussion

One of the main challenges identified by this study among infertile women who live in the city of Bichena was the psychological challenges. These women have described their psychological challenges through stress, anxiety, depression, low self-esteem, and sexual dissatisfaction. The findings of this study indicated that stress frequently affects infertile women and that they have experienced it in various ways. According to the findings, the main ways infertile women express stress due to infertility problems include crying, forgetfulness, disturbance, and lack of sleep. The same findings were presented by different studies that are mentioned in a review of related literature.^[1,2,12,19]^

To validate this example, Simionescu et al^[23]^ investigated that infertile couples are subject to greater stress and are at increased risk of developing psychological disorders compared to healthy normal couples. The study also mentioned that women have experienced more psychological stress than men due to cultural issues. Ahinkorah et al^[18]^ also ensured that the stress experienced may arise from a multitude of factors such as the desire to have children and possess motherhood and the strains imposed on the marital relationships. Similarly, in this study the findings indicate that infertile women are stressed due to the finding of children, fear of losing marital status, and the breakdown of the relationship with the husband’s relatives.

Furthermore, Liang et al^[7]^ clearly found that women with infertility have experienced stress and accompanied by different stress symptoms such as inability to sleep, excessive crying, palpitations, and lack of concentration. This finding is consistent with this study except palpitation, which is not a major symptom of infertility stress for infertile women in Bichena town. Another finding of this study in relation to psychological challenges is anxiety. The finding has shown that infertile women in the town of Bichena have experienced anxiety and describe it in different ways. The findings indicated that thinking too much, frustration, and doubtfulness are the main means of expressing anxiety by infertile women in the study area. The finding of this study is similar to the study conducted by Iordachescu et al^[24]^ regarding infertility related to infertility, which investigated that infertile women are highly vulnerable to experiencing anxiety. The study also revealed that women have higher rates of anxiety levels associated with infertility and report infertility as one of the most stressful experiences in their life. However, the level of anxiety is not measured in this study due to the nature of the approach of this study. More studies are needed to measure the level of anxiety.

Additionally, Akelewold^[11]^ found that anxiety is one of the psychological problems that occur among infertile women. Jemberu and Yemane^[12]^ also find that infertile women have often thought about their problems and fear of their future lives. This is consistent with the anxiety components identified by this study, which are too much thinking and frustration for infertile women. Furthermore, Lawali^[6]^ studied infertile women in Nigeria and investigated the same findings in this study. Depression is another psychological problem identified by this study among infertile women in the city of Bichena. According to the findings of this study, infertile women in the study area have experienced sadness, unhappiness, and a feeling of anger. The study investigated by Akelewold^[11]^ has validated the prevalence of depression symptoms among infertile women. Halkola et al^[4]^ also stated the same finding that women with infertility problems are suffering with sadness, anger, and grief.

Another psychological challenge identified by this study among infertile women is low self-esteem. The finding of this study has revealed that infertile women in the study area have very low respect and give little value for themselves compared to fertile women because they are unable to bear children. This study finding is validated by Liang et al^[7]^ which stated that infertile women’s value of infertile women’s current social relationships deteriorates and they cannot meet the relationship standards of another person as they avoid social inclusion, and in turn, society can isolate them due to cultural and social stigmas. Moreover, Zhang et al^[25]^ pointed out that when infertile women were exposed to stigma (social challenges) that highly contribute and can lower their self-esteem and self-efficacy. The findings of this study also indicated that infertile women in the study area feel sexual dissatisfaction due to infertility-related problems. To support this finding, Akalewold^[11]^ confirmed that infertile women have experienced sexual dissatisfaction and felt the loss of marital and social status by their spouses.

Studies have indicated that in almost all societies, infertile women experience a certain level of stigmatization depending on the varied social structures. In Africa, infertile individuals are inclined to hide their status of infertility from community for fear of social stigma. Higher rates of fear of social exclusion in infertile women are resulting from infertility. For example, in Ghana, women are reported to have social stigma, marital stress, instability, and social isolation^[6]^; in Zambia by Howe et al.^[26]^ The main social components that cause psychological challenges were isolation, stigma, family and social pressure, marital instability, and low social status.^[19,26]^

Infertile women were found to use different coping strategies, such as religious strategies, traditional strategies, medical strategies, and other strategies (marital separation, distancing, and acceptance). Similarly, infertile women have used different religious activities to cope with their infertility challenge.^[4,10,11,27,28]^ For example, Lawali and Abbas^[27]^ revealed that most Ghanaian women use religious faith to cope with infertility. Odek et al^[28]^ proved that infertile women in Kenya, Kisumu County, became churchgoers and attended the church as a very different person. Honestly, they have personally seen the hand of God, especially through fellowship. They believe that God is faithful and that 1 day they will get a healthy baby boy. This study cleared the same finding that infertile women in the town of Bichena have preferred religious strategies and believed in God to receive his miracle of giving children.

Another important finding of the coping strategies used by infertile women in the study area was medical strategies. As the study found that some infertile women have tried to solve their infertility problem through medical treatments. However, some of them stopped the treatment due to financial deficit and time consuming. To support this finding, Hiadzi et al^[29]^ stated that infertile women often used assisted reproductive technology (ART).

Another finding of this study is that infertile women in the study area went to different wizards and traditional healers to solve their infertility problems. Previous studies released similar findings.^[11,13,28,30]^ For example, Akalewold^[11]^ found that some infertile women went to wizards to solve their infertility problems and have practiced unwanted sexual intercourse with male wizards. The same finding obtained in this study was that some infertile women were exposed to having sexual intercourse with wizards without their conscious awareness. Infertile women are also pressured by wizards to continue and stay at the wizard’s house, otherwise the wizard sanctions them. In addition to these parts of the plants, traditional herbalists in the town of Bichena use the roots of the plant, the blood of the plant (liquid) and the cover of the plant (skin). Traditional healers used different plants for infertile women and women. Validating this, Ofosu-Budu and Hänninen^[30]^ reported that Ghanaian herbalists did not use the same amount and type of plant medicine for both sexes, there are plants that allow infertile women and other different plants for infertile men. This study also found that traditional herbalists used different parts of the plant by mixing and squeezing all together. This is consistent with the Jaradat and Zaid^[13]^ finding that traditional herbalists in the West Bank use whole plants believing that they are more beneficial than isolated plant constituents.

Another coping strategy used by infertile women in the study area is marital separation. Most infertile women reported that they feel a great pressure from various sources to end their marriage. Some infertile women have not resisted the pressure and terminated their marriage and prefer to remain single. Similarly, previous studies such as Akalewold,^[11]^ Lawali, and Abbas^[27]^ stated that infertile women were divorced due to infertility-related challenges when they experience social pressure or family pressure. This study also told that some infertile women in the study area have experienced longer years of waiting for their pregnancy and have tried different ways to conceive. However, they couldn’t. Therefore, they prefer to accept their problem and if a miracle happened, they wait. Similar findings were published by Howe et al,^[26]^ Ofosu-Budu and Hänninen^[30]^ that infertile women finally accept their infertility problem, either they reached the menopause stage or they were found to withdraw from medical treatment due to a prolonged period of medical treatment and perceived less benefits of that treatment. These little variations in terms of strategies used to overcome psychological challenges could be brought about by variations in the research year, shifts in community attitudes, and variations in the study environment. This study is supported by Scheper-Hughes and Lock’s three-body approach which stated that the individual body experiences health, happiness, or sorrow are influenced by social, political, and cultural factors.

### 4.1. Conclusion, implications and future directions

To sum up the above discussion, infertile women in Bichena town are challenged by many aspects. They have suffered psychological challenges in the form of stress, anxiety, depression, low self-esteem, and sexual dissatisfaction. Infertile women also reported that they have used different coping strategies to solve their problems. In this study, spiritual (religious) strategies, traditional strategies, medical strategies, informal foster care, marital separation, and accepting their problems were identified.

This study also has theoretical implications for the current literature, knowledge and experience, and practical implications. The following recommendations are made based on the findings of the study with the following bodies: Infertile women of Bichena town, Ministry of Education, Bichena hospital administrative management bodies, Non-governmental organizations, counselors and researchers in developmental psychology and other interested investigators. Infertile women in Bichena city have tested different forms of coping strategies in combination. However, the researchers recommended that they do not go to wizards who need to have unwanted sexual intercourse with the born baby. Such a spirit has no scientific evidence according to this study and previous work studies. They shall also consult with professionally trained counselors and psychiatrists in nearby hospitals to reduce stress, anxiety, and depression related to infertility. The community members should respect and treat both infertile women and men equally and prepare some guideline to support infertile women through different types and phases of medical treatment for which financial deficits have faced.

This study was also limited to one town, Bichena town; thus, the study can be expanded by including various towns and districts, where cultural influences on psychological challenges and coping strategies of infertile women may or may not differ. However, the study will serve as a base for other scholars to study the current issue. Some respondents can have biased views due to their cultural background or personal views that affect the study findings.^[25]^ A more objective result will be reached by using multiple research instruments or scales, such as depression, stress, or additional psychological conditions in the local context, together with the participation of several respondents. As a result, quantitative measurement will allow future researchers to investigate the existing problem.

Furthermore, with a descriptive phenomenological design, by collecting data at one time, it is less likely to infer the general psychological challenges and coping mechanisms of infertile women. Therefore, future scholars would use longitudinal study designs to provide more conclusive and substantiate information. This article does not cover the views of infertile men. The study would be carried out using various data collection sources using men as a survey and reference.

## Acknowledgments

We thank infertile women for participating in this investigation.

## Author contributions

**Conceptualization:** Tinisaie Biadigie Adane, Kelemu Zelalem Berhanu, Abatihun Alehegn Sewagegn.

**Data curation:** Tinisaie Biadigie Adane, Kelemu Zelalem Berhanu, Abatihun Alehegn Sewagegn.

**Formal analysis:** Tinisaie Biadigie Adane, Kelemu Zelalem Berhanu, Abatihun Alehegn Sewagegn.

**Methodology:** Tinisaie Biadigie Adane, Kelemu Zelalem Berhanu, Abatihun Alehegn Sewagegn.

**Supervision:** Tinisaie Biadigie Adane, Kelemu Zelalem Berhanu, Abatihun Alehegn Sewagegn.

**Validation:** Abatihun Alehegn Sewagegn.

**Writing – original draft:** Tinisaie Biadigie Adane, Kelemu Zelalem Berhanu, Abatihun Alehegn Sewagegn.

**Writing – review & editing:** Tinisaie Biadigie Adane, Kelemu Zelalem Berhanu, Abatihun Alehegn Sewagegn.
